# Scientific Items

**Published:** 1880-12-20

**Authors:** 


### SCIENTIFIC ITEMS.

**Copying Ink.**—Copying ink is made from many of the ordinary by the addition of sugar or glycerine, which prevent them from becoming perfectly dry. When a page written with this ink is moistened, the ink becomes sufficiently liquid to allow an impression to be taken from it on thin unsized paper.—*Boston Journal of Chemistry.*

**Why the Needle Points to the North.**—Prof. C. T. Paterson, of the United States Coast Survey, in reply to an inquiry, wrote the following clear and concise explanation of the *directive* action of the magnetic needle :

The reason why the needle points in the northernly direction is that the earth in itself is a magnet, attracting the magnetic needle as the ordinary magnets do; and the earth is a magnet as the result of certain cosmical facts, much affected by the action of the sun. These laws have periodicities, all of which have not as yet been determined.

A condensed explanation in regard to the needle pointing to the northward and southward is as follows : The magnetic poles of the earth do not coincide with the geographical poles. The axis of rotation makes an angle of about 23° with a line joining the former.

The northern magnetic pole is at present near the arctic circle on the meridian of Omaha. Hence the needle does not everywhere point to the astronomical north, and is constantly variable within certain limits. At San Francisco it points about 17° to the east of north, and at Calais, Maine, as much to the west.

At the northern magnetic pole a balanced needle points with its north end downward in a plumb line; at San Francisco it dips about 63°, and at the southern magnetic pole the south end points directly down.

The action of the earth upon a magnetic needle at its surface is of about the same force as that of a hard steel magnet forty inches long, strongly magnetized, at a distance of one foot.—*Ibid.*

**Lunar Geology.**—J. Landerer has submitted to the Paris Academy a work in which he seeks to determine the lithologic character of our satellite. He thinks that the density of the moon and the angle under which it polarizes the light of the sun are such as to show that the materials of the surface are analogous to those of the silicate rocks.

**An Electro-Magnetic Experiment.**—According to Nature, Signor Agostini finds that if through a drop of mercury, lying on a surface not wet by it, a current of electricity be sent in a vertical direction it rotates under the influence of the earth's magnetism, as may be seen if a few particles of lycopodium powder be strewn on it. Similarly a mercury drop rotates when it is placed on the surface of a steel magnet, and the magnet is connected with a positive pole of a very weak element, while an electrode penetrating the drop from above is connected with the negative. From the strength and direction of rotation of a number of such drops, one may make visible the distribution of the magnetism in the magnetic bars themselves, as when an iron bar is brought coaxially near to one end or into contact; also in the latter. The results of previous experimental measurements are thus confirmed. —*Boston Journal of Chemistry*.

**The Blood of Intermittents.**—Dr. E. L. Moss, staff surgeon in the British Navy, has constantly found, after the lapse of forty-eight or more hours, organisms in the blood of intermittent fever which he was unable to find in fresh blood. The organisms consist of bacteria, singly or in pairs, in active movement, sometimes stationary in zo-oglea groups, occasionally in chains of four or more. Dr. Moss's method would appear to exclude every possibility of infection.

If further investigation should confirm Dr. Moss's discovery, it will add greatly to our means of differentiation of fevers, and may lead to important therapeutic results.—*Medical Herald*.

**A New Battery.**—MR. REYNIER has introduced a new battery which is claimed to possess twice the electro-motive force of the ordinary Bunsen couple; a solution of caustic soda is substituted for nitric acid, and the "porous" cell is constructed of parchment paper.

**A New Lens.**—Dr. Cusco, of Paris, has invented a lens of variable focus, in which the pressure of transparent liquid is made to alter the curvature of the flat faces of a cylindrical cell of brass closed with thin glass discs; the pressure can be regulated by a manometer gauge to any required degree within the limits of working.

**Atoms.**—Some important discoveries of tin have been made near Thornborough, Queensland; it assayed, it is said, 74 to 76 per cent.

A LACUSTRINE village, rich in flint implements, and other relics of the age of stone, has been discovered near Gerlafingen, on the lake of Neuchatel.

THE electric light has been applied to illuminate a volcano; there are nine lights on Mount Vesuvius.

M. PASTEUR has received the sum of $8,000 from the French Government to enable him to continue his researches on the contagious diseases of animals.

DRS. DE LA RUE and MULLER determine the height at which the aurora borealis has its greatest brilliancy as about 38 miles; at a height of 81 miles the light is pale and faint; and at 124 miles above the surface, no electric discharge can take place to produce the phenomenon.

THE turbine wheel of a foundry at New Edinburgh (Ontario) was lately stopped three times by the enormous number of eels going down the river.

DR. WRIGHT, a pupil of the Philadelphia doctor factory, recently gave a burial certificate in the case of a child, setting forth that it had died of "collary fantum."

BENSON gave this sage advice to his friends: "In house accommodation live above your means, in clothing up to your means, and in food below your means."

